# A Novel Diagnostic and Treatment Approach to an Unusual Case of Dens Invaginatus in a Mandibular Lateral Incisor Using CBCT and 3D Printing Technology

**DOI:** 10.3390/dj12040107

**Published:** 2024-04-13

**Authors:** Lindsey LaLonde, Mazin Askar, Susan Paurazas

**Affiliations:** Graduate Endodontics, University of Detroit Mercy School of Dentistry, Detroit, MI 48208, USA; lalondld@udmercy.edu (L.L.); askarma@udmercy.edu (M.A.)

**Keywords:** dens invaginatus, CBCT, 3D printing, 3D models, large foramen, bioceramics, platelet-rich fibrin, mineral trioxide aggregate, endodontic surgery

## Abstract

Background: This case report demonstrates the use of three-dimensional (3D) models produced from a cone beam computed tomographic (CBCT) volume to develop a treatment strategy for a rare type of dens invaginatus (DI) in a mandibular incisor. Methods: A patient with DI Type IIIa presented for endodontic treatment. Following CBCT evaluation, the complex morphologic nature of the invagination required additional diagnostic tools for treatment planning. The fabrication of 3D models provided clarity regarding the treatment strategy. Treatment involved intracanal medication with calcium hydroxide Ca(OH)_2_, nonsurgical root canal therapy (NS-RCT) of the main canal, and endodontic surgery for the DI anomaly using mineral trioxide aggregate (MTA), bone graft, and platelet-rich fibrin (PRF) membrane. Results: The use of 3D models provided an invaluable guide for proper treatment. Complicating factors were diagnosed and planned for accordingly. Conclusions: It is difficult to appreciate the anatomical complexity, the extent, and the nature of the invagination of rare Type III DI morphology. CBCT imaging and 3D models played a critical role in the pre-treatment planning to ensure a predictable outcome. A 3D model is recommended as a diagnostic tool in treating complex cases where the DI morphology is wide, oblique, or the foraminal opening is irregular.

## 1. Introduction

Dens invaginatus is a unique developmental malformation resulting in an enamel lined cavity intruding into the crown and/or root. When evaluating the tooth to determine what type of DI was present, references to case reports in the literature proved to be useful. The most commonly accepted classification proposed by Oehlers for DI formations are Types I, II, IIIa, and Type IIIb [1]. When identifying the developmental anomaly associated with the current case, fusion was ruled out since there were no missing teeth in the dental arch and clinical presentation was consistent with Type IIIa descriptions. Findings were consistent with the diagnosis of DI as described by Zhu, which includes a barrel shaped tooth and dilated crown with increased mesiodistal diameter, which was present in the treated tooth [2]. According to Gallacher, referring to Oehler’s classification of DI, Type I is confined within the crown. Type II extends beyond the CEJ without periodontal ligament communication. In Type IIIa, the invagination extends through the root and communicates laterally with the periodontal ligament space through a pseudo-foramen. This morphology is consistent with the current case. In Type IIIb, the invagination extends through the root and communicates with the periodontal ligament at the apical foramen [3]. Type I makes up 79% of cases, 15% of cases are Type II, and the rarest type of DI is Type III with 6% of cases [4]. The clinical presentation of the current DI case also had the same characteristics as DI Type III, as profiled in several case reports [5,6,7].

Type IIIa formations pose particular challenges because of the highly variable nature of their apical opening and the location of the pseudo-foramen opening [8,9,10,11]. Their foramens range from small to large and they can have open, oblique architectures. In smaller, flatter foramens, an apexification procedure can be used with either a collagen barrier or PRF as an apical plug followed by MTA backfilling. DIs with wide, oblique foramina pose a significant risk of underfilling or overfilling from the nonsurgical treatment approach. These cases necessitate endodontic surgery to properly clean, disinfect, and control the use of biomaterials [12,13]. The biocompatibility of materials like bioceramics at the interface of the foraminal exit and the bone is ideal [14]. The purpose of this case report is to demonstrate the use of CBCT and 3D printing technologies to properly plan and facilitate treatment strategies to treat a mandibular lateral incisor (#26) with a rare Type IIIa DI. To our knowledge, there is one other DI case report documenting the benefits of 3D models, and two case reports for DI Type IIIa in mandibular incisors [7,15,16]. The present case demonstrates the benefits provided by CBCT and 3D models in the treatment planning of a very wide and oblique architecture of the anatomical anomaly. This approach provided the clinician with a clear strategy to reach a satisfactory treatment outcome.

## 2. Materials and Methods

**Figure 1:**
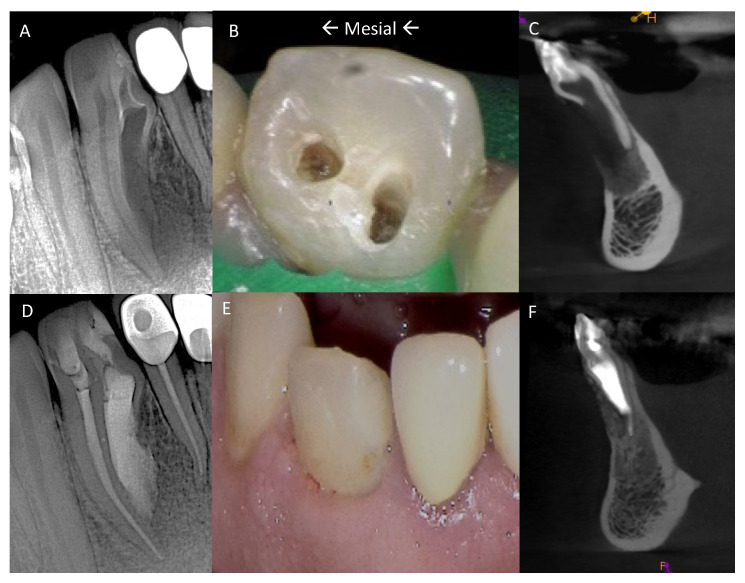
(**A**) Initial PA taken at endodontic evaluation of tooth #26. (**B**) Mesial and distal endodontic access cavities. (**C**) Pretreatment CBCT sagittal section. (**D**) Final PA after completion of nonsurgical and surgical endodontic treatments and restoration. (**E**) Photograph of restored tooth #26. (**F**) Post-treatment CBCT at 6-month follow-up.

**Figure 2:**
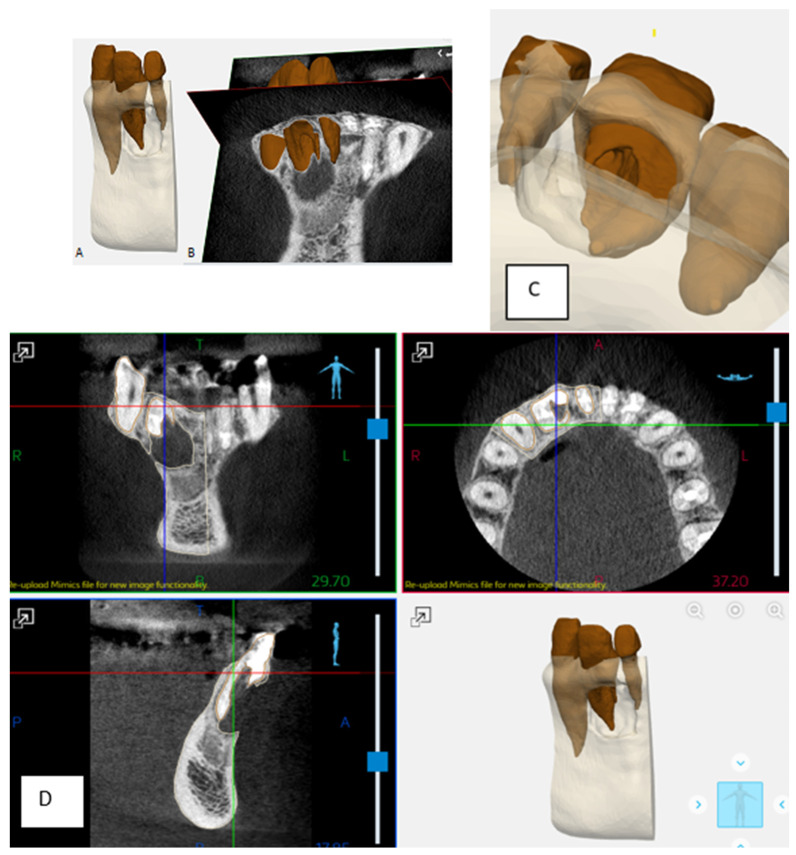
Renderings used during the design process. (**A**,**C**) Teeth #25–#27 within the alveolus. Both the DI and the lesion are modeled. (**B**) Teeth #25–#27 with overlaid axial and coronal CBCT sections. (**D**) CBCT imaging used to design 3D tooth model with mapping of the DI and surrounding tissues.

**Figure 3:**
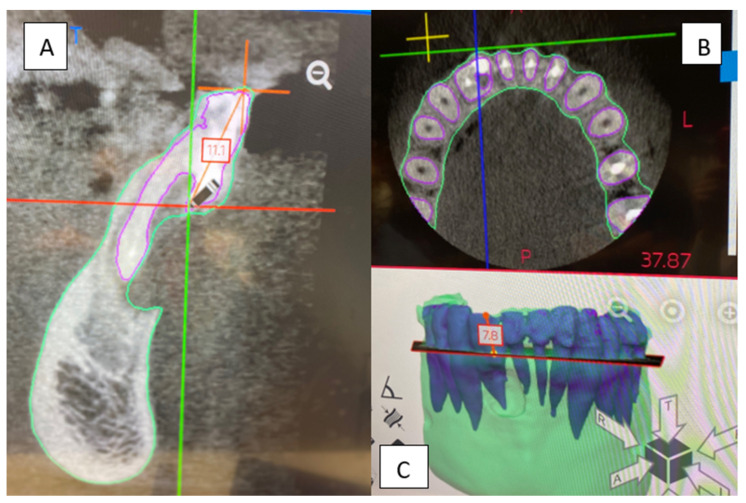
CBCT sagittal (**A**) and axial (**B**) sections of tooth #26. (**C**) Measurements taken from the CBCT sections and 3D software aided in accurate non-surgical and surgical planning. Axial section apically showing dilated space of DI.

**Figure 4:**
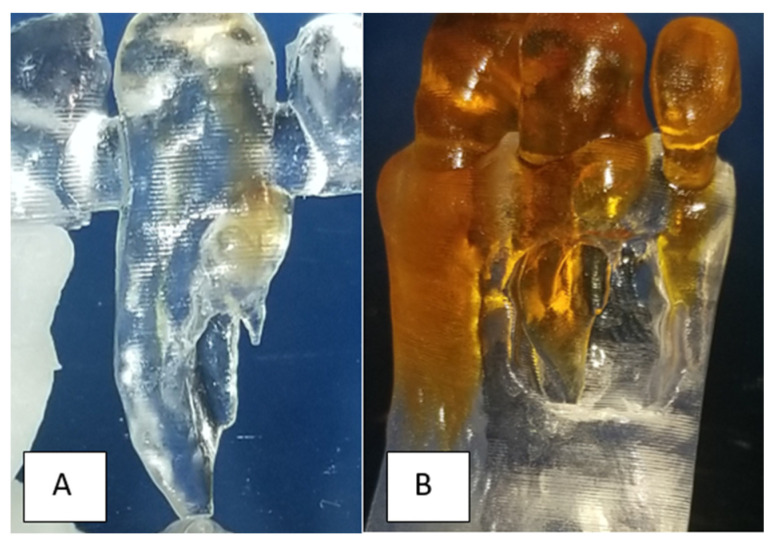
Images of both the 3D models. (**A**) Tooth #26 alone. Light brown color traces the DI pathway. Moving apically, it begins in a mesial pathway and turns distally at the midroot. (**B**) Teeth #25–#27 within the alveolus used for surgical planning.

**Figure 5:**
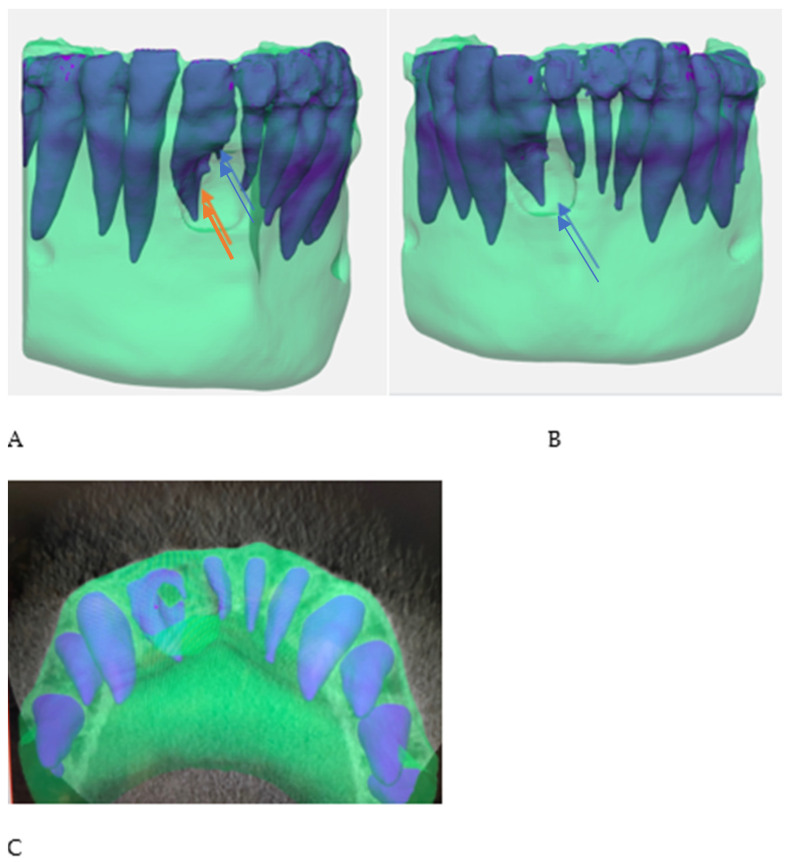
3D jaw models utilized in pre-surgical planning. 3D model showing the mesiofacial projection: (**A**) (blue arrow) the lingual wing; (**A**) (orange arrow) apical aspect of the DI with lingual wing formation; (**B**) extent of periapical bony lesion; and (**C**) axial view representation.

A 32-year-old female was referred to the Graduate Endodontic Clinic at the University of Detroit Mercy School of Dentistry for evaluation and treatment of a mandibular right lateral incisor. The patient’s health history was noncontributory. Extraoral examination was unremarkable. Intraoral examination revealed a fixed prosthesis from #23–#24 and a metal–ceramic crown on #25. Tooth #26 was wider in a mesiodistal dimension relative to the adjacent crowns. There was a mesial composite restoration. There was no pain to percussion or palpation, no mobility, no response to cold or electric pulp testing, and periodontal probing was 2–3 mm circumferentially. Periapical radiographs showed two canal spaces. The main distal canal had a normal shape with a completely formed root apex. The mesial invaginated portion was consistent with a rare type of DI Type IIIa. The invagination had a wide opening extending obliquely along more than half the length on the mesial wall of the distal root (Figure 1). There was a periapical lesion involving #26 and #25. Dimensions of the lesion on CBCT measured 25.2 × 15 × 12.4 mm. The endodontic diagnosis was pulpal necrosis with asymptomatic apical periodontitis. Due to the extensive nature of the periapical pathology, the patient was seen previously by an oral surgeon for biopsy 11 months prior to the endodontic evaluation. The specimen was most consistent with an inflammatory cyst. Based upon the DI complexity, further analysis was needed for a clearer understanding of the morphology. A digital rendering was utilized for measurements of critical structures and to plan the creation of 3D models (Figure 2 and Figure 3). Two 3D models were produced from Materialise (Plymouth, MI, USA). The first 3D model replicated the tooth itself and the second model exhibited the location and orientation of the tooth within the alveolus (Figure 4). The software packages used were Materialise Mimics Medical 23.0 (Materialise NV, Leuven, Belgium) and Materialise 3-matic Medical 15.0 (Materialise NV, Leuven, Belgium). At the crestal bone level, the canal turned distally at a 45-degree angle, opened widely along the adjacent root surface, and continued as part of the major foramen. The complex morphology of the DI foramen opening was bordered by three major anatomical dentinal structures: a mesiofacial projection, a lingual wing forming most of the invaginated structure, and the main axial root surface of the distal root. The lingual wing of the root in the coronal plane extended beyond the DI foramen nearly to the level of 2 mm from the root apex (Figure 4 and Figure 5). The wing terminated at a subtle notch apically. The mesiofacial projection acted as a partition extending 5 mm in the apical direction from the most coronal portion of the apical opening. The 3D model allowed us to see a subtle depression nearly to length in the axial root surface. The arrangement of these structures resembled a half-tube formation with a very wide opening. 

### 2.1. Pretreatment Planning 

**Figure 6:**
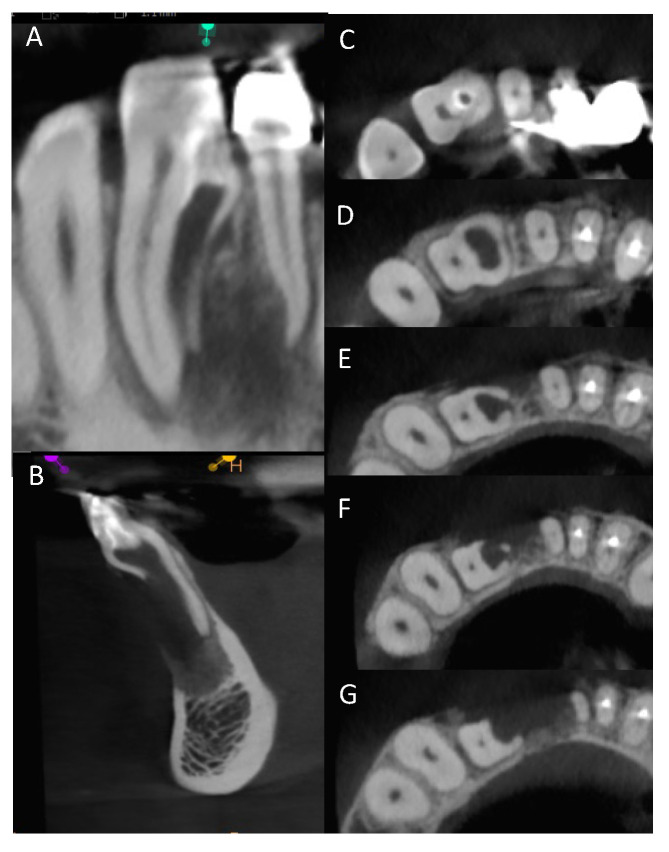
(**A**) CBCT coronal section of tooth #26. (**B**) CBCT sagittal section. (**C**) Coronal axial section with both chamber spaces. (**D**) Axial section apically within the coronal third showing dilated space of DI. (**E**) Axial section midroot area with lateral DI foramen to the mesial. (**F**) Axial section in apical third showing the mesiofacial projection and the lingual wing. (**G**) Apical aspect of DI with lingual wing formation.

The 3D models and the CBCT volume were crucial to pretreatment planning. The 3D models demonstrated that adequate orthograde access was not possible when treating the DI portion. The nature of the angulated portion of the DI would necessitate excessive coronal and cervical dentin removal within the crown and compromise long-term prognosis (Figure 4 and Figure 6). Accordingly, the decision was made to plan a surgical approach for the DI. The main distal canal was planned for nonsurgical root canal treatment (NS-RCT) through conservative access. A second, smaller access over the DI was planned to allow for Ca(OH)_2_ before the surgical procedure (Figure 1). 

The next phase of planning addressed the disinfection protocol and sealing of the DI surgically. The half-tube extending from the apical third of the root to the DI posed a risk of retaining microbes [17]. It was planned for passive ultrasonic irrigation followed by placement of MTA to seal the entire length of the depression extending into the coronal portion of the DI. The size of the lesion and its apical–coronal extension were additional concerns for presurgical planning. The DI was planned for long-term calcium hydroxide medication placement to disinfect and to control the microbial load in the defect, with the goal of reduction in lesion size [18,19,20]. PRF placement was planned to provide advantages in osteopromotion, for prevention of epithelial downgrowth along the root surface, and to preserve the coronal epithelial and gingival tissue attachment level. One PRF membrane layer would secure the MTA on the half-tube during the initial setting period, preventing dislodgment during the grafting process. A second membrane would be placed over the entire grafted defect. The final flap design, specifically the width and placement of vertical incisions, was determined using the second 3D model (Figure 5). Both 3D models allowed for pretreatment troubleshooting and working through these separate issues until a single, cohesive treatment plan was developed. Treatment would use a combined treatment approach of nonsurgical root canal treatment (NS_RCT) in the main distal canal and endodontic surgery for the DI portion. Instrumentation, disinfection, and sealing from the retrograde approach would conserve tooth structure and allow for more accurate placement of materials. The 3D models were invaluable in the pre-surgical planning to determine the extent of MTA placement and the subsequent plan to use PRF to protect the retrofilling during the grafting procedure.

### 2.2. Nonsurgical Treatment

The area of tooth #26 was anesthetized with 1.7 mL of 4% articaine with 1:100,000 epinephrine (Dentsply Pharmaceutical, York, PA, USA) with local infiltration and a rubber dam isolation applied. Two access openings were made on the lingual surface of the tooth into the main distal canal and the DI portion. Recurrent caries was identified and removed. Patency was established in the main distal canal using a size 10 Kfile (Lexicon Presterilized, Dentsply Sirona, Johnson City, TN, USA) and working length was determined using an electronic apex locator (J. Morita Corp, Kyoto City, Japan) and verified with a periapical radiograph. Measurements obtained from the CBCT and the models allowed us to estimate a safe working length range for irrigation and Ca(OH)_2_ placement (Figure 3). A 10 mL luer-lock syringe (Becton, Dickinson and Company, Franklin Lakes, NJ, USA) with a side-vented, 30-gauge tip (ProRinse, Dentsply Tulsa Dental Specialties, Johnson City, TN, USA) filled with CHX-Plus 2% Chlorhexidine Gluconate Solution (Vista Dental Products, Racine, WI, USA) was used for irrigation due to concern about possible accidental extrusion into the periradicular tissues. The main distal canal was instrumented to a size 30, taper 0.04 with an Edge X7 file (Edge-Endo, Albuquerque, NM, USA). UltraCal XS (Ultradent Products, South Jordan, UT, USA) was used as intracanal medication. The periods between intracanal medications were irregular due to COVID-19 restrictions. In total, six medication appointments occurred during a six-month period. A cotton pellet and Fuji IX (GC America Inc, Alsip, IL, USA) were used for temporization between visits. After the medication period, another CBCT scan was taken to evaluate current changes. Since the CBCT from 18 months prior to the start of treatment, the lesion reduced to 11.3 × 10 × 5 mm in largest dimensions. At the final visit and before surgery, the main distal canal was obturated using Conform Fit Gutta Percha (Dentsply Sirona, Johnson City, TN, USA) and EndoSequence BC Sealer (Brasseler, Savannah, GA, USA). Fuji IX (GC America, Alsip, IL, USA) was placed in the DI access. The main distal canal access was restored with A2-B Premise composite (Kerr, Orange, CA, USA). The patient was scheduled for the surgical treatment of the DI portion.

### 2.3. Surgical Treatment

**Figure 7:**
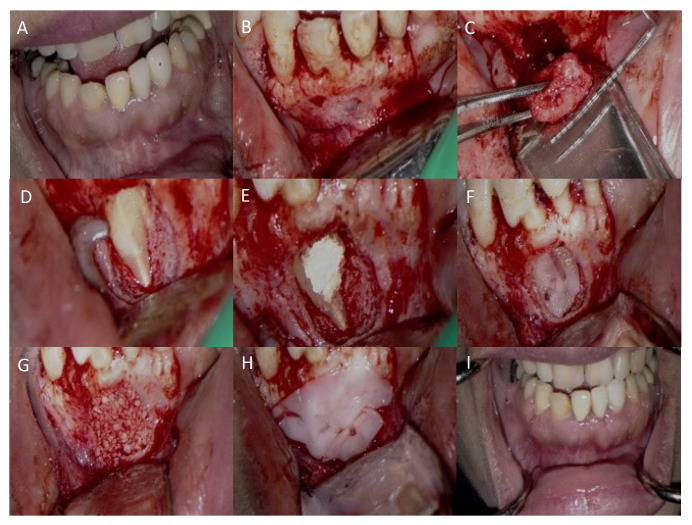
The surgical treatment sequence. (**A**) Presurgical field. (**B**) Flap reflection and lesion identification. (**C**) Lesion removal. (**D**) DI and the mesiofacial projection. (**E**) MTA retrofilling placement. (**F**) PRF membrane placement over MTA. (**G**) Grafting particulate in place. (**H**) PRF membrane placement over the grafting site. (**I**) One-week post-operative photograph.

**Figure 8:**
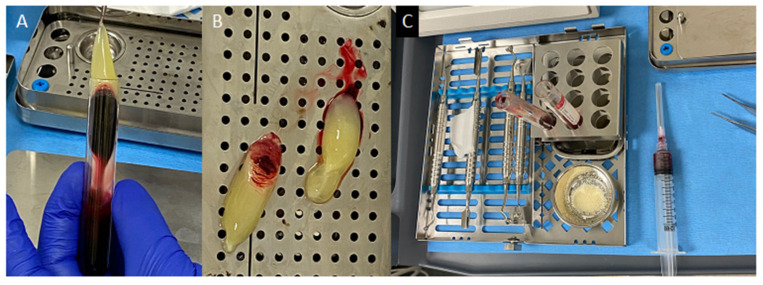
(**A**) PRF removal from 10 mL test tube. (**B**) Two PRF layers. (**C**) Materials prepared for grafting. Allograft particulate to be mixed with red corpuscle.

The patient reported one week later for surgery (Figure 7). The surgical site was anesthetized with 1.7 mL of 2% lidocaine with 1:50,000 epinephrine (Dentsply Pharmaceutical, York, PA, USA) around the root tips of teeth # 25,26,27. The remaining surgical areas were anesthetized with 3.4 mL of 2% lidocaine HCl with 1:100,000 epinephrine (Dentsply Pharmaceutical, York, PA, USA). A full thickness mucoperiosteal flap with two vertical incisions at the distal of #24 and the distal of #27 was reflected. The lesion was visualized within the cortical bone defect. A single, complete specimen of a firm, fibrous tissue was curetted from the bone measuring 10 × 6 × 5 mm (Figure 7C). The underlying #26 tooth structure could then be visualized. The lingual wing was readily observed continuing coronally to the oblique, open tube of the invagination. The remnants of Ca(OH)_2_ were rinsed thoroughly and removed using a 10 mL syringe of sterile saline and a side-vented, size 30 irrigation tip (ProRinse, Dentsply Tulsa Dental Specialties, Johnson City, TN, USA). Passive ultrasonic irrigation (PUI) with a KiS-1D Ultrasonic tip (Obtura Spartan Endodontics, Algonquin, IL, USA) was used within the tubular portion of the invagination to eliminate attached debris. ProRoot MTA (Dentsply Sirona, Johnson City, TN, USA) was placed and contoured to seal the DI part coronally and extended apically to fill the remaining portion of the DI along the adjacent root surface depression. Blood was drawn from the patient’s antecubital fossae, filling two 10 mL test tubes without anticoagulant. One step centrifugation at 2700 rpm (750 g) for 12 min produced blood product separation. Two PRF scaffolds were produced and pressed between a perforated and a non-perforated metal plate to create two membranes (Figure 8). The first membrane was placed over the MTA and retrofilling Puros^R^ particulate allograft particles (1.0 cc, Zimmer, Alachua, FL, USA) were mixed with 2 mL of the patient’s blood and placed into the defect (Figure 7F). The second PRF membrane was placed over the entire defect. (Figure 7H). The flap was re-approximated and sutured with six interrupted 5-0 Prolene sutures (Ethicon, San Lorenzo, PR, USA). Postsurgical instructions were given to the patient verbally and in writing. A follow-up examination and suture removal appointment were scheduled. 

## 3. Results

**Figure 9:**
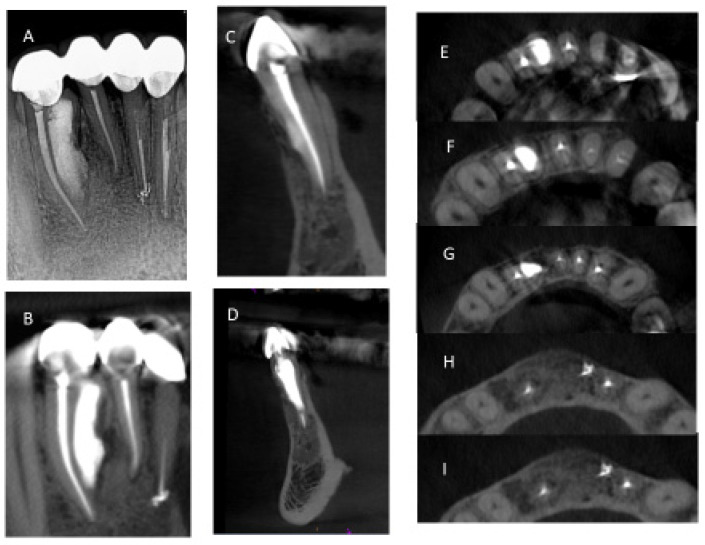
Post-treatment CBCT at 36 month follow-up showed bony healing on 2D image (**A**) and on small FOV CBCT coronal section (**B**), sagittal section (**C**,**D**), and axial sections (**E**–**I**).

During the first week of follow-up, healing proceeded normally with minimal discomfort. At 6 weeks, the mesial access cavity was restored with Premise Composite A1-B and A2-B (Kerr, Orange, CA, USA). Radiographic and clinic follow-ups at 1, 3, 6, and 36 months showed clinical success and radiographic evidence of healing. At the 6-month follow-up, CBCT imaging confirmed complete healing (Figure 1D–F). At the 3-year follow-up appointment, the patient was asymptomatic. Splinted crowns had been placed on teeth 23–26. Periodontal probing depths were less than or equal to 3 mm. There was no sensitivity to palpation, percussion, or bite testing. A CBCT image was exposed and showed complete bony healing. (Figure 9).

## 4. Discussion

Endodontic success depends on proper case selection, accurate diagnosis, and precise treatment planning [21,22]. The treatment outcome of teeth with DI, especially those associated with apical periodontitis, involves a more comprehensive approach to achieving adequate disinfection and sealing quality. The use of the CBCT and the production of 3D models contributed significantly to our understanding of the 3D nature of the complex morphology of DI, the surrounding tissue defect, and proper treatment management. The use of the two 3D models at the pretreatment planning stage were of great value to determining the necessary treatment protocol for nonsurgical treatment of the main distal canal and surgical treatment for the DI. The use of 3D printed models from CBCT for endodontic teaching purposes has previously been described [23]. With the unique challenges associated with this rare and complex DI Type IIIa identified before treatment, the treatment team prepared the patient properly for the selected treatment strategy. All underlying structures were known and planned for accordingly. The procedure was executed efficiently and led to healing without incident. The 3D model accurately simulated the clinical presentation upon flap reflection and access to the surgical area (Figure 5). It is worth noting that achieving this kind of accuracy depended on coordination with the software engineer. The engineer works on detail to the level of the voxel in the designing process. Careful interpretation of the CBCT between the endodontist and the software engineer is essential to creating a 3D model that provides the maximum benefit to presurgical planning and to facilitate the surgical procedure itself (Figure 2, Figure 3, Figure 4 and Figure 5). The use of tooth models has been profiled in a case report supporting the use of 3D models to address an anomalous complex anatomy [24] and improve workflow and add objectivity to the treatment planning process [25].

1. The comparison between the use of 3D printing and milling with the use of Materialise software, as used in the current study, was profiled in a study by Kachhara et al. They concluded that an exact tooth replica can be created to assist in treatment planning [26]. The ability of the clinician to view a 3D model versus manipulating a CBCT image in the axial, sagittal, and coronal views, is beneficial to appreciating the fine detail of the unique root anatomy. The exact replica is produced to a high level of detail that can be directly viewed. Being able to handle the physical model provides an opportunity to manipulate and observe the model and appreciate the unique characteristics of the tooth and root anatomy when conceptualizing the proposed nonsurgical and surgical endodontic treatment.
2. Potential challenges to using 3D printing technology for treatment planning include access to technology, knowledge of appropriate software, cost, and patient acceptance of complex treatment plans. When using advanced technologies, patients should be informed of potential complications. When considering the use of advanced technology, the benefit to the patient should be of primary concern. Materials used in this case have been extensively researched, such as MTA and advanced platelet rich fibrin (A-PRF). The unique aspect of this case is the use of combined therapies with advanced 3D printing technology to ensure a more predictable, inclusive treatment. Longer term follow-up, as seen in this case, is encouraged to continue to evaluate the successful outcome of treatment in rare, unusual cases.
3. The use of two PRF membranes made several significant contributions to treatment execution. The soft, flexible nature of PRF is ideal for layering over MTA without adversely affecting the surface texture. The first membrane delicately smoothed and contained the material within the confines of the DI during the setting period. The second PRF membrane was used to protect the entire defect and to retain the grafting particulate and maintain its osteoconductive properties, enabling the ingrowth of vascular and cellular connective tissues. The PRF in grafting gives the advantage of using the patient’s blood products to maximize available growth factors and to dramatically improve the speed of osseous healing [8,27,28]. For DIs with a wide, oblique foramen, PRF is critical to retaining and protecting the MTA filling while providing the above-mentioned healing benefits. The present case report demonstrated that using 3D models led to improved pretreatment planning and provided a more accurate protocol for the surgical intervention.

## 5. Conclusions

Fabrication of 3D models from a limited field of view CBCT as part of treatment planning for rare cases of DIs with wide, oblique foramina and complex morphology ensures the proper treatment protocol, thereby avoiding potential treatment complications. Understanding the available nonsurgical access limitations led to proper surgical planning and a more conservative access strategy. The models facilitated the surgical planning. Accurate diagnostics led to quality treatment planning and a successful combined nonsurgical and surgical endodontic treatment.

## Data Availability

Restrictions apply to the availability of these data. Data were obtained from Materialise and are available with the permission of Materialise.
